# Real‐World Usage of a Paclitaxel‐Coated Balloon With Urea Compared With Other Contemporary Drug‐Coated Balloons: A 2‐Year Analysis From SCAAR in Over 6000 Patients

**DOI:** 10.1161/JAHA.125.045621

**Published:** 2026-05-29

**Authors:** Sacharias von Koch, Erika Frank, Katja Gabrysch, Stefan James, Matthias Götberg, Moman A. Mohammad, David Erlinge

**Affiliations:** ^1^ Department of Cardiology, Clinical Sciences Lund University, Skåne University Hospital Lund Sweden; ^2^ Uppsala Clinical Research Center Uppsala University Uppsala Sweden; ^3^ Department of Medical Sciences Uppsala University Uppsala Sweden

**Keywords:** drug‐coated balloons, paclitaxel, percutaneous coronary intervention, Revascularization, Percutaneous Coronary Intervention

## Abstract

**Background:**

Drug‐coated balloons (DCB) are frequently used during percutaneous coronary intervention. Despite this there are limited data from studies comparing different types of DCB, particularly for a paclitaxel‐coated balloon (PCB) using urea as the excipient. This study aimed to assess the clinical efficacy of the PCB with urea compared with other frequently used DCB, attempting to emulate a target trial using observational data.

**Methods:**

SCAAR (Swedish Coronary Angiography and Angioplasty Registry) is a nationwide registry including data on all patients in Sweden undergoing coronary angiography. For this study, all procedures with DCB in Sweden between August 2021 and May 2024 were included. Propensity score weighted multivariable Cox regression analysis and Kaplan–Meier estimates were used to assess outcomes through 2 years.

**Results:**

A total of 1797 (27.7%) procedures with the PCB with urea and 4691 (72.3%) procedures with Other DCB were eligible for this analysis. DCB treatment was performed in a variety of patients and lesions including 32.4% diabetes, 64.2% acute coronary syndrome, 20.5% bifurcations and 33.0% in‐stent restenosis. After 2 years, the event rates for the PCB with urea and Other DCB were 16.4% versus 18.4% for major adverse cardiovascular events (hazard ratio [HR] 1.01 [95% CI 0.84–1.22]), 7.2% versus 8.2% for all‐cause mortality (HR 1.05 [95% CI 0.80–1.39]), 6.1% versus 8.0% for myocardial infarction (HR 0.81 [95% CI 0.59–1.11]), 14.6% versus 14.9% for new revascularization with percutaneous coronary intervention (HR 1.02 [95% CI 0.83–1.25]), 0.8% versus 1.3% for target lesion definite thrombosis (HR 0.93 [95% CI 0.41–2.11]), 7.4% versus 8.7% for target lesion revascularization (HR 0.95 [95% CI 0.70–1.29]), and 10.5% versus 11.3% for target vessel revascularization (HR 0.99 [95% CI 0.76–1.29]).

**Conclusions:**

In this analysis evaluating 2‐year clinical outcomes after DCB, in a broad patient population, the PCB with urea had comparable outcomes with other contemporary DCB. This data further support the safety and efficacy of the PCB with urea.

## Graphical abstract

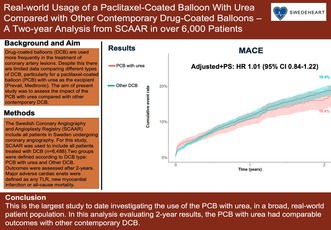

**Nonstandard Abbreviations and Acronyms**
DCB: drug‐coated balloon
MACE: major adverse cardiovascular events
PCB: paclitaxel‐coated balloon
SCAAR: Swedish Coronary Angiography and Angioplasty Registry
SCB: sirolimus‐coated balloon

**Clinical Perspective**

**What Is New?**

- In this all‐comer analysis, paclitaxel‐coated balloon with urea was associated with a similar outcome compared with other contemporary drug‐coated balloon (DCB).
- This study encompasses 1797 procedures with the paclitaxel‐coated balloon with urea, making it the largest study to investigate the use of this device and, to the best of our knowledge, with 6488 total DCB procedures the largest study to date comparing the use of different types of DCB.

**What Are the Clinical Implications?**

- Previous studies have shown that there are differences in clinical outcome among DCB and, given this, these data are important for clinicians using DCB in their clinical practice.

Despite the implementation of drug‐eluting stents, in‐stent restenosis continues to pose a significant challenge following percutaneous coronary intervention (PCI).^1^ In light of this, drug‐coated balloons (DCB) have emerged as a treatment alternative to drug‐eluting stents.

There are a variety of DCBs available today differing in their drug type (paclitaxel and sirolimus), coating technology and balloon design. While some have found no discernible differences when comparing different types of DCB,^2^, ^3^, ^4^ an observational study comparing the use of 2 paclitaxel‐coated balloons (PCB) found a significant difference in incidence of restenosis at 6 months [3.4% versus 12.5%; adjusted risk ratio: 0.39; 95% CI (0.24–0.65)].^5^ The results of this study suggest there are clinical differences in outcomes depending on DCB design and that factors other than the drug likely affect clinical outcomes. Considering this, data comparing assorted types of DCB are warranted.

A PCB using urea as the excipient (Prevail, Medtronic) is currently one of the most frequently used DCB in Sweden.^6^ A small prospective single‐arm trial that included 50 patients treated with the PCB with urea found that treatment of coronary de novo, small vessel, and in‐stent restenosis lesions resulted in low late lumen loss at 6 months and low rates of revascularization and safety events through a 12‐month follow‐up period.^7^ While there is an ongoing pivotal trial assessing the PCB with urea, the clinical evidence beyond the aforementioned pilot trial remains limited despite its wide adoption.^8^ In contemporary Swedish practice, DCBs have become more commonly used across centers. Selection between urea‐coated PCB and other DCB platforms is primarily determined by device availability, local practice patterns, and operator preference related to deliverability and experience. This study attempts to emulate a target trial using observational data with the aim to evaluate the 1‐ and 2‐year outcomes when using the PCB with urea compared with other frequently used contemporary DCB in a real‐world clinical practice in Sweden (Table S1).^9^

## METHODS

### Study Design

We conducted a retrospective observational study using SCAAR (Swedish Coronary Angiography and Angioplasty Registry).^10^ SCAAR is a comprehensive nationwide registry including all patients undergoing coronary angiography in any of the 29 hospitals in Sweden that provide acute cardiac care. The registry includes >200 variables including patient characteristic (eg, age, sex, comorbidities), lesion characteristics (eg, bifurcations, in‐stent restenosis) and procedural information (eg, type of DCB, balloon size). As SCAAR is a quality care registry no consent is needed for the inclusion in the registry, all patients are informed about the registry and have the option to opt out. SCAAR is continuously linked with the National Population Registry to update survival status. This linkage enables a comprehensive analysis of all patients undergoing PCI in Sweden, with virtually complete follow‐up for all the patients. The study was approved by the Swedish Ethical Review Authority (Dnr 2023‐00201‐01) and research was performed in accordance with appropriate ethical guidelines. Because of the sensitive nature of the data collected for this study, requests to access the data set from qualified researchers trained in human subject confidentiality protocols may be sent to Uppsala Clinical Research Center. The study protocol was not registered.

### Study Population and Outcome

**Figure 1:**
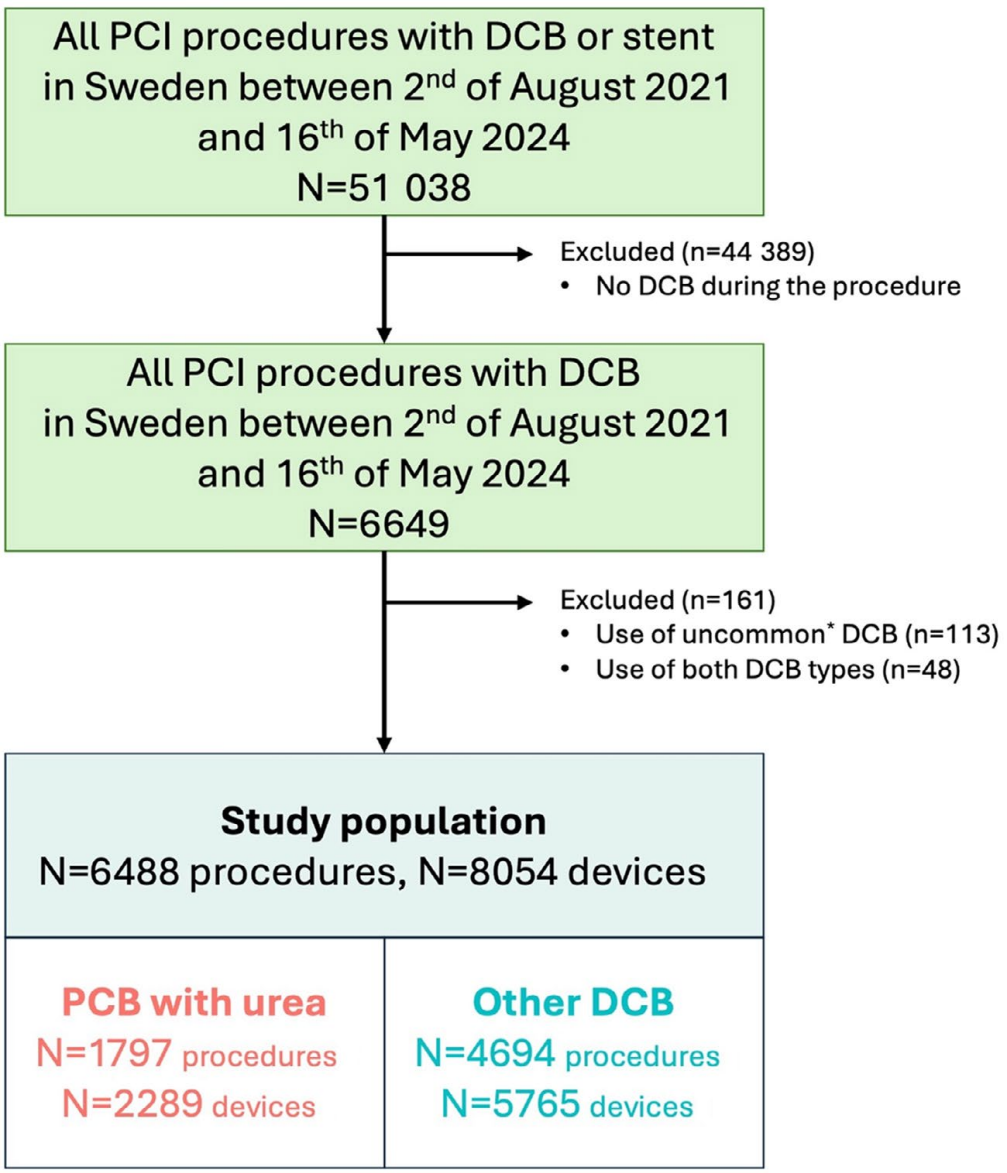
Flow chart. Study flow chart illustrating inclusion and exclusion criteria. The final study population comprised of 6488 procedures with 8054 DCB. DCB indicates drug‐coated balloons; PCB, paclitaxel‐coated balloon; and PCI, percutaneous coronary intervention. *Uncommon DCB refers to DCB used <100 times during the study period.

For this study, all procedures utilizing a DCB in SCAAR were included between August 2, 2021 and May 16, 2024 (Figure 1). Exclusion criteria comprised uncommon DCB which were used <100 times during the study period and procedures in which both the PCB with urea and other DCB were used. Two groups were formed based on type of DCB: *the PCB with urea* and *Other DCB*. The PCB with urea (Prevail, Medtronic) was introduced to the Swedish market August 2, 2021, which defined the start of the study period. This balloon uses urea as an excipient and paclitaxel as an antirestenotic drug with a target dose of 3.5 μg/mm^2^. A list of DCB devices included in the Other DCB group is presented in Table 1. Outcomes were assessed on device‐level and procedure‐level. Outcomes assessed in the device‐level analysis comprised target lesion definite thrombosis, target lesion revascularization and target vessel revascularization. The procedure‐level analysis included all‐cause mortality, new myocardial infarction, any revascularization with PCI and major adverse cardiovascular events (MACE). MACE was defined as the composite of any TLR, new myocardial infarction or all‐cause mortality. Definitions of outcomes are in Table S2. All outcomes were clinically driven and were evaluated at 1‐ and 2‐years post‐PCI as available in our national registry up to May 16, 2024. While not all patients reached every follow‐up point, data were complete through the ascertainment date with no losses to follow‐up.

**Table 1:** Other DCB Definition

Name	Drug	Excipient	Number of devices
Braun SeQuent® Please	Paclitaxel	Iopromide	3322
Boston Scientific Agent™	Paclitaxel	Acetyl tributyl citrate	1124
Biotronik Pantera® Lux	Paclitaxel	Butyryl‐tri‐hexyl citrate	1030
Cordis Selution SLR™	Sirolimus	Multiple excipients	289
Total			5765

### Statistical Analysis

Baseline characteristics were collected including patient demographics, procedural information, and angiographic findings. Continuous variables are presented as medians and quartiles, and categorical data as counts with percentages. Outcomes were analyzed using univariable and multivariable Cox regression analysis along with Kaplan–Meier estimates. The multivariable analysis was adjusted for the following variables: age, sex, diabetes, hypertension, hyperlipidemia, previous myocardial infarction, previous PCI, previous coronary artery by‐pass graft surgery, indication (chronic coronary syndrome, unstable angina, non–ST‐segment elevation myocardial infarction, ST‐segment elevation myocardial infarction and other indications), angiographic findings (non‐conclusive, no angiographically significant stenosis, 1 vessel disease, 2 vessel disease, 3 vessel disease and left main disease), bifurcation, chronic total occlusion, in‐stent restenosis, B2/C lesions (according to American heart association classification), use of intravascular ultrasound, use of optic coherence tomography, DCB diameter and use of stent during PCI. Propensity scores were calculated using logistic regression including all variables listed above. The resulting scores were used in the unadjusted and adjusted Cox regression through inverse probability weighting. The hazard assumption was verified for the Cox regression using residual plots (Figure S1). A directed acyclic graph of the variables included in the adjustment model is presented in Figure S2. No colliders, mediators, or instrumental variables were included in the adjusted model. Age was entered into the model as a restricted cubic spline with 3 knots. Both regression models were conducted on complete case data as the proportion of missing values in variables of interest were low (Table 2). The Huber sandwich estimator was used to handle the dependence between patients with multiple procedures as well as patients undergoing PCI with several DCBs. Results from the Cox regression analysis are presented as hazard ratios along with 95% CIs and *P* values. The results from the Kaplan–Meier analysis are presented as number of events and failure estimates. Subgroup analyses were conducted for diabetes, acute coronary syndrome, de novo lesions, in‐stent restenosis, small vessel (DCB diameter ≤2.75 mm), and use of stent during the procedure and bifurcations. Subgroups were analyzed using the adjusted models after 2 years, and for the outcomes target lesion revascularization, myocardial infarction, all‐cause mortality and MACE. Subgroups were assessed individually together with *P*‐value of interactions. In addition to this adjusted analysis, number of events and Kaplan–Meier estimates after 1 and 2 years are also presented. In a sensitivity analysis, the Other DCB group was divided into Other PCB group and sirolimus‐coated balloon (SCB) group. For this sensitivity analysis, we excluded procedures in which both Other PCB and SCB were used. The 3 groups were assessed using Kaplan Meier estimates and log rank test. Because of the lack of randomization, all analyses relied on identifying assumptions in relation to the observational data, including conditional exchangeability, positivity, and consistency across the variables. A 2‐sided *P* value <0.05 was considered statistically significant. Data management and all statistical analyses were conducted in R (The R Foundation for Statistical Computing, Vienna, Austria).

**Table 2:** Baseline Characteristics

Procedure characteristics	PCB with urea	Other DCB	Missing
	N=1797	N=4691	(n)
Age	70.1 (61.8–77.9)	72.2 (63.2–78.5)	0
Women	399 (22.2%)	1025 (21.9%)	0
Smoking status: Non‐smoker	756 (45.6%)	2025 (45.3%)	360
Previous smoker	706 (42.6%)	1920 (42.9%)	
Current smoker	195 (11.8%)	526 (11.8%)	
Diabetes: Yes, insulin treated	240 (13.4%)	695 (14.9%)	27
Yes, not insulin treated	328 (18.3%)	820 (17.6%)	
Yes, unknown treatment	3 (0.2%)	14 (0.3%)	
No	1218 (68.1%)	3143 (67.3%)	
Hyperlipidemia	1287 (71.8%)	3454 (73.9%)	22
Hypertension	1416 (79.0%)	3805 (81.4%)	20
Creatinine (μmol/L)	83.0 (70.2–100.0)	83.0 (70.0–99.0)	351
BMI (kg/m^2^)	26.9 (24.4–29.8)	27.1 (24.5–30.1)	438
Previous MI	805 (45.0%)	2254 (48.5%)	49
Previous PCI	1002 (55.8%)	2816 (60.0%)	1
Previous CABG	191 (10.6%)	480 (10.2%)	1
Indication: Chronic coronary syndrome	434 (24.2%)	1092 (23.3%)	0
Unstable angina	224 (12.5%)	554 (11.8%)	
NSTEMI	671 (37.3%)	1849 (39.4%)	
STEMI	202 (11.2%)	663 (14.1%)	
Other	266 (14.8%)	533 (11.4%)	
Angiographical finding: Non‐conclusive	1 (0.1%)	8 (0.2%)	1
Atheromatosis	98 (5.5%)	363 (7.7%)	
1‐vessel not LM	752 (41.9%)	1980 (42.2%)	
2‐vessel not LM	562 (31.3%)	1283 (27.4%)	
3‐vessel not LM	281 (15.6%)	725 (15.5%)	
LM	102 (5.7%)	332 (7.1%)	
Successful procedure*	1774 (98.8%)	4647 (99.1%)	2

Device/lesion characteristics	PCB with urea	Other DCB	Missing
	N=2289	N=5765	(n)
Stent in same vessel	1108 (48.4%)	2559 (44.4%)	0
IVUS used in same segment	411 (18.0%)	734 (12.7%)	0
OCT used in same segment	121 (5.3%)	391 (6.8%)	0
IVL used in same segment	34 (1.5%)	115 (2.0%)	0
Atherectomy used in same segment	0 (0.0%)	0 (0.0%)	0
Local success†	2174 (98.7%)	5634 (98.4%)	132
Balloon length (mm)	20.0 (20.0–30.0)	20.0 (15.0–30.0)	16
Balloon diameter (mm)	2.5 (2.2–3.0)	2.5 (2.2–3.0)	6
Balloon diameter (mm): <2.5 mm	635 (27.8%)	1624 (28.2%)	6
2.5–3.0 mm	1238 (54.2%)	2904 (50.4%)	
>3.0 mm	411 (18.0%)	1236 (21.4%)	
Lesion type: De novo	1629 (71.2%)	3665 (63.6%)	0
Other restenosis	19 (0.8%)	82 (1.4%)	
In‐stent restenosis	641 (28.0%)	2018 (35.0%)	
B2/C	1265 (55.3%)	2932 (50.9%)	0
Occlusion/CTO: Chronic total occlusion	75 (3.3%)	206 (3.6%)	9
Acute total occlusion	162 (7.1%)	564 (9.8%)	
No occlusion	2050 (89.6%)	4988 (86.6%)	
Treated vessel: RCA	447 (19.5%)	1134 (19.7%)	0
LM	36 (1.6%)	129 (2.2%)	
LAD	1166 (50.9%)	2780 (48.2%)	
LCx	600 (26.2%)	1546 (26.8%)	
Artery graft	6 (0.3%)	3 (0.1%)	
Vein graft	34 (1.5%)	173 (3.0%)	
Bifurcation	521 (22.8%)	1134 (19.7%)	0

Presented as median (Q1–Q3) or n (%).

BMI indicates body mass index; CABG, coronary artery bypass graft; DCB, drug‐coated balloons; IVL, intravascular lithotripsy; IVUS, intravascular ultrasound; LAD, left anterior descending; LCx, left circumflex artery; LM, left main; MI, myocardial infarction; NSTEMI, non–ST‐segment elevation myocardial infarction; OCT, optical coherence tomography; PCB, paclitaxel‐coated balloon; PCI, percutaneous coronary intervention; RCA, right coronary artery; and STEMI, ST‐segment elevation myocardial infarction.

* Successful procedure. Subjective assessment by the operator. The operator has reached the main aim of the treatment.

^†^ Treated stenosis of 50% or greater, with a reduction of at least 20% leading to a final stenosis degree of less than 50%, accompanied by good flow and no major complication.

### Target Trial Emulation Framework

Analyses were conducted within a target trial emulation framework. The hypothetical randomized trial was specified with assignment to treatment strategies at baseline, prespecified outcome definitions, and follow‐up extending to 1 and 2 years after treatment initiation. These components were emulated in the observational data by defining comparable treatment strategies, outcomes, and follow‐up periods. Causal effects were estimated through adjustment for baseline covariates measured prior to treatment initiation. In the absence of randomization, valid inference relied on standard identifying assumptions for observational analyses, including conditional exchangeability, positivity, and consistency across the variables.

## RESULTS

### Study Population Characteristics

A total of 8054 DCB and 6488 procedures were eligible. The PCB with urea comprised 2289 (28.4%) of the DCB and 1797 (27.7%) of the procedures (Table 2). The Other DCB group comprised of 5438 (95.6%) PCB and 253 (4.4%) SCB (Table S3). For the PCB with urea and Other DCB respectively, the mean age was 70.1 and 72.2 years, 22.2% and 21.9% were women, 31.9% and 32.8% had diabetes, and 45.0% and 48.5% had a previous myocardial infarction. Among the patients treated with the PCB with urea and Other DCB, 61.0% and 65.4% had acute coronary syndrome and 52.6% and 49.9% had multivessel disease and/or left main disease. Of the lesions that were treated with the PCB with urea and Other DCB, 55.3% and 50.9% were B2/C lesions, 28.0% and 35.0% were in‐stent restenosis, 22.8% and 19.7% were bifurcations, 3.3% and 3.6% were chronic total occlusions, and 27.8% and 28.2% had a balloon diameter <2.5 mm (Table 2). Intracoronary imaging was used in 27.1% of the in‐stent restenosis lesions and 14.5% of the de‐novo lesions.

### Outcome

**Figure 2:**
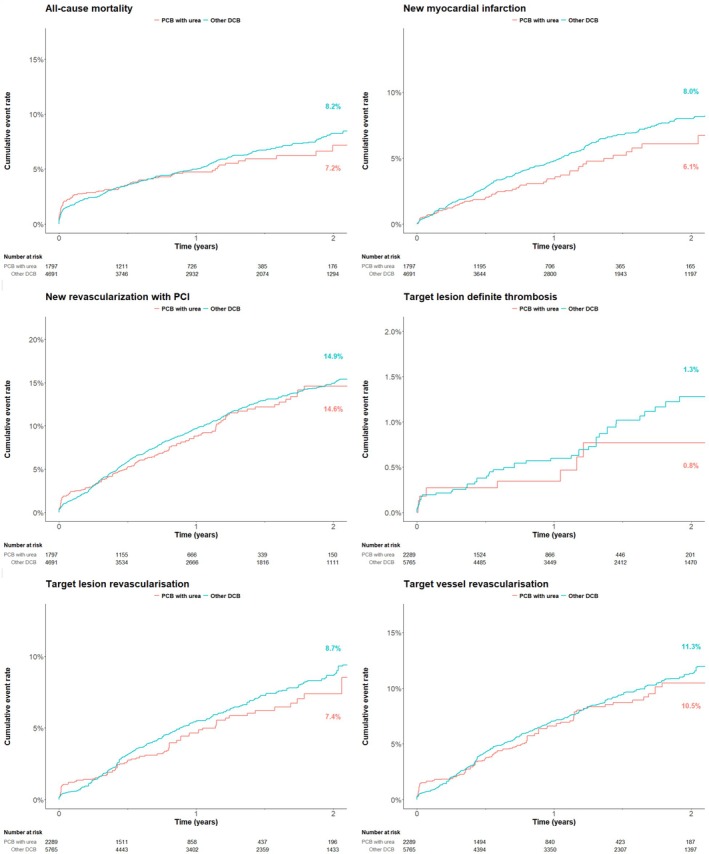
Kaplan–Meier estimates. Illustrating the estimated event rates of all‐cause mortality, new myocardial infarction and new revascularization with PCI for patients treated by the PCB with urea (red, N=1797 procedures) and Other DCB (blue, N=4691 procedures); and target lesion definite thrombosis, target lesion revascularization and target vessel revascularization (N=2289 PCB with urea, N=5765 Other DCB devices). DCB indicates drug‐coated balloons; PCB, paclitaxel‐coated balloon; and PCI, percutaneous coronary intervention.

After use of the PCB with urea and Other DCB, the proportion of local success was 98.7% and 98.4% (Table 2). After adjusting for confounders and propensity score, after 1 year for the PCB with urea and Other DCB respectively, the estimated event rates were 4.7% versus 5.0% for all‐cause mortality [HR 1.12 (95% CI 0.83–1.51)], 3.4% versus 4.8% for myocardial infarction [HR 0.77 (95% CI 0.54–1.10)], 8.8% versus 9.7% for new revascularization with PCI [HR 0.97 (95% CI 0.77–1.22)], 0.3% versus 0.6% for target lesion definite thrombosis [HR 0.82 (95% CI 0.29–2.27)], 4.6% versus 5.5% for target lesion revascularization [HR 0.92 (95% CI 0.66–1.28)], 6.6% versus 7.1% for target vessel revascularization [HR 0.95 (95% CI 0.71–1.27)], and 10.6% versus 11.5% for MACE [HR 1.04 (95% CI 0.85–1.27)] (Figure 2; Table 3).

**Table 3:** Outcome

	PCB with urea	Other DCB	Unadjusted	Adjusted*	PS†	Adjusted*+PS†
			HR (95% CI)	HR (95% CI)	HR (95% CI)	HR (95% CI)
1‐y outcome						
MACE	152 (10.6%)	649 (11.5%)	0.95 [0.78–1.16] *P* value: 0.602	1.06 [0.87–1.29] *P* value: 0.566	1.03 [0.84–1.26] *P* value: 0.755	1.04 [0.85–1.27] *P* value: 0.722
All‐cause mortality	72 (4.7%)	292 (5.0%)	0.98 [0.74–1.29] *P* value: 0.869	1.11 [0.83–1.48] *P* value: 0.493	1.11 [0.83–1.48] *P* value: 0.479	1.12 [0.83–1.51] *P* value: 0.448
New myocardial infarction	45 (3.4%)	244 (4.8%)	0.70 [0.49–1.00] *P* value: 0.050	0.80 [0.56–1.13] *P* value: 0.204	0.76 [0.53–1.10] *P* value: 0.144	0.77 [0.54–1.10] *P* value: 0.152
New revascularization with PCI	116 (8.8%)	519 (9.7%)	0.91 [0.72–1.14] *P* value: 0.400	0.97 [0.78–1.22] *P* value: 0.806	0.96 [0.76–1.20] *P* value: 0.696	0.97 [0.77–1.22] *P* value: 0.776
Target lesion definite thrombosis	7 (0.3%)	39 (0.6%)	0.66 [0.26–1.66] *P* value: 0.378	0.75 [0.30–1.92] *P* value: 0.552	0.78 [0.28–2.16] *P* value: 0.637	0.82 [0.29–2.27] *P* value: 0.698
Target lesion revascularization	76 (4.6%)	349 (5.5%)	0.89 [0.64–1.23] *P* value: 0.468	0.97 [0.70–1.34] *P* value: 0.849	0.90 [0.65–1.25] *P* value: 0.521	0.92 [0.66–1.28] *P* value: 0.611
Target vessel revascularization	108 (6.6%)	462 (7.1%)	0.94 [0.70–1.26] *P* value: 0.681	1.01 [0.76–1.34] *P* value: 0.958	0.93 [0.70–1.25] *P* value: 0.639	0.95 [0.71–1.27] *P* value: 0.731
2‐y outcome						
MACE	179 (16.4%)	809 (18.4%)	0.93 [0.78–1.12] *P* value: 0.450	1.04 [0.86–1.24] *P* value: 0.704	[0.84–1.22] *P* value: 0.893	1.01 [0.84–1.22] *P* value: 0.881
All‐cause mortality	82 (7.2%)	366 (8.2%)	0.93 [0.71–1.22] *P* value: 0.604	1.06 [0.81–1.39] *P* value: 0.679	1.04 [0.79–1.37] *P* value: 0.773	1.05 [0.80–1.39] *P* value: 0.725
New myocardial infarction	58 (6.1%)	317 (8.0%)	0.73 [0.54–0.99] *P* value: 0.046	0.82 [0.60–1.12] *P* value: 0.213	0.80 [0.58–1.09] *P* value: 0.163	0.81 [0.59–1.11] *P* value: 0.190
New revascularization with PCI	142 (14.6%)	635 (14.9%)	0.95 [0.78–1.16] *P* value: 0.638	1.02 [0.83–1.24] *P* value: 0.864	[0.82–1.24] *P* value: 0.915	1.02 [0.83–1.25] *P* value: 0.837
Target lesion definite thrombosis	10 (0.8%)	56 (1.3%)	0.73 [0.34–1.53] *P* value:0.402	0.86 [0.40–1.88] *P* value: 0.711	0.85 [0.38–1.89] *P* value: 0.690	0.93 [0.41–2.11] *P* value: 0.860
Target lesion revascularization	91 (7.4%)	432 (8.7%)	0.90 [0.67–1.20] *P* value: 0.475	0.99 [0.74–1.33] *P* value: 0.955	0.94 [0.70– 1.26] *P* value: 0.682	0.95 [0.70–1.29] *P* value: 0.761
Target vessel revascularization	129 (10.5%)	573 (11.3%)	0.95 [0.73–1.23] *P* value: 0.707	1.03 [0.80–1.34] *P* value: 0.807	0.97 [0.75–1.26] *P* value: 0.836	0.99 [0.76–1.29] *P* value: 0.960

DCB indicates drug‐coated balloons; HR, hazard ratio; MACE, major adverse cardiovascular events; PCB, paclitaxel‐coated balloon; and PCI, percutaneous coronary intervention.

n (%*P*) represents total events (Kaplan‐Meier estimate).

* Adjusted for: age, sex, diabetes, hypertension, hyperlipidemia, previous myocardial infarction, previous PCI, previous coronary artery by‐pass graft surgery, indication (chronic coronary syndrome, unstable angina, non–ST‐segment elevation myocardial infarction, ST‐segment elevation myocardial infarction and other indications), angiographic findings (non‐conclusive, no significant stenosis, 1 vessel disease, 2 vessel disease, 3 vessel disease and left main disease), bifurcation, chronic total occlusion, in‐stent restenosis, B2/C lesions (according to American heart association classification), use of intravascular ultrasound, use of optic coherence tomography, DCB diameter and use of stent during PCI.

^†^ Propensity scores were calculated using a logistic regression including all variables listed above. The resulting scores were used in the Cox regression through inverse probability weighting.

After adjusting for confounders and propensity score, after 2 years for the PCB with urea and Other DCB respectively, the estimated event rates years were 7.2% versus 8.2% for all‐cause mortality [HR 1.05 (95% CI 0.80–1.39)], 6.1% versus 8.0% for myocardial infarction [HR 0.81 (95% CI 0.59–1.11)], 14.6% versus 14.9% for new revascularization with PCI [HR 1.02 (95% CI 0.83–1.25)], 0.8% versus 1.3% for target lesion definite thrombosis [HR 0.93 (95% CI 0.41–2.11)], 7.4% versus 8.7% for target lesion revascularization [HR 0.95 (95% CI 0.70–1.29)], 10.5% versus 11.3% for target vessel revascularization [HR 0.99 (95% CI 0.76–1.29)], and 16.4% versus 18.4% for MACE [HR 1.01 (95% CI 0.84–1.22)].

### Subgroup and Sensitivity Analysis

**Figure 3:**
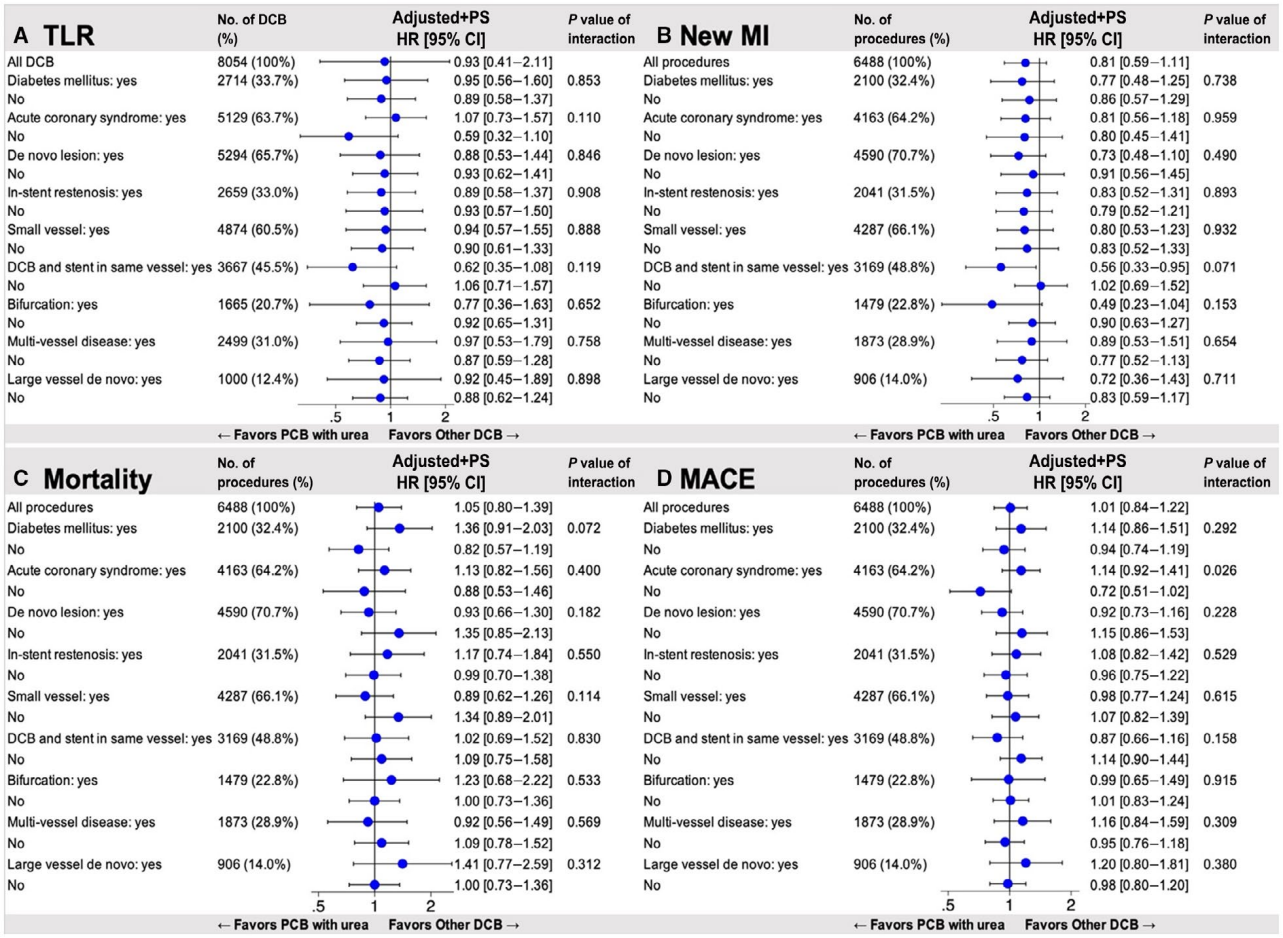
Subgroup analysis. Subgroups were assessed after 2 years for (**A**) target lesion revascularization and (**B**) new myocardial infarction, (**C**) all‐cause mortality and (**D**) major adverse cardiovascular events (MACE) using an adjusted [Adjusted for: age, sex, diabetes, hypertension, hyperlipidemia, previous myocardial infarction, previous PCI, previous coronary artery by‐pass graft surgery, indication (chronic coronary syndrome, unstable angina, non–ST‐segment elevation myocardial infarction, ST‐segment elevation myocardial infarction and other indications), angiographic findings (non‐conclusive, no significant stenosis, 1 vessel disease, 2 vessel disease, 3 vessel disease and left main disease), bifurcation, chronic total occlusion, in‐stent restenosis, B2/C lesions (according to American heart association classification), use of intravascular ultrasound, use of optic coherence tomography, DCB diameter and use of stent during PCI] Cox proportional hazards model with PS (PS were calculated using a logistic regression including all variables listed above. The resulting scores were used in the Cox regression through inverse probability weighting) used through inverse probability weighting. DCB indicates drug‐coated balloons; HR, hazard ratio; MACE, major adverse cardiovascular events; PCB, paclitaxel‐coated balloon; PCI, percutaneous coronary intervention; and PS, propensity scores.

A subgroup analysis investigated the 2‐year outcome for the PCB with urea and Other DCB for diabetes, acute coronary syndrome, de novo lesion, in‐stent restenosis, small vessel, DCB and stent in same vessel, bifurcation, multi‐vessel disease, and large vessel de novo (Figure 3). No statistically significant difference was observed concerning target lesion revascularization, new myocardial infarction, all‐cause mortality or MACE between the PCB with urea and Other DCB for patients with diabetes, acute coronary syndrome, de novo lesions, in‐stent restenosis, small vessels (DCB diameter ≤2.75 mm), bifurcations, multi‐vessel disease or large vessel de novo. For patients treated with DCB and stent in the same vessel (hybrid approach), the PCB with urea was associated with lower rates of new myocardial infarction [HR: 0.56 (95% CI: 0.33–0.95)]. No difference was observed for target lesion revascularization, all‐cause mortality or MACE for patients treated with DCB and stent in the same vessel. A statistically significant treatment‐by‐subgroup interaction was observed in terms of MACE for the subgroup acute coronary syndrome. Number of events and Kaplan–Meier estimates for subgroups are presented in Table S4.

In the sensitivity analysis comparing the PCB with urea with Other PCB and sirolimus DCB (SCB), the estimated 2‐year event rate was 7.2% versus 8.3% versus 4.9% for all‐cause mortality, 6.1% versus 7.8% versus 18.4% for myocardial infarction, 14.6% versus 14.6% versus 21.1% for new revascularization with PCI. For device level outcomes the estimated event rates through 2 years were 0.8% versus 1.3% versus 2.0% for target lesion definite thrombosis, 7.4% versus 7.9% versus 22.1% for target lesion revascularization, and 10.5% versus 10.6% versus 23.2% for target vessel revascularization, respectively (Figure S3). Finally, when comparing the PCB with urea with the individual other PCB, the results were in line with the main outcome showing no statistical difference in outcome (Table S5; Figure S4).

## DISCUSSION

We conducted a nationwide real‐world assessment of the use of a PCB with urea, one of the most used DCBs. In this all‐comer analysis, the PCB with urea was associated with a similar result compared with other frequently used contemporary DCB. This study comprises of 1797 procedures with a PCB with urea, making it the largest study to investigate the use of this device and, to the best of our knowledge, with 6488 DCB procedures the largest study to date comparing the use of different types of DCB. Given the differences in outcomes among the various DCB designs, these data are important for clinicians using DCB in their clinical practice.

The implementation of drug‐eluting stents has significantly reduced the risk of restenosis compared with bare‐metal stents.^11^ Despite improved treatment strategies, in‐stent restenosis continues to pose a significant challenge comprising 10% of all PCI and is associated with a significantly worse outcome compared with de novo lesions.^1^, ^12^ The European Guidelines emphasize DCB in the treatment of in‐stent restenosis,^13^ recommendations that are based mainly on data from an iopromide excipient‐based PCB. The excipient is of importance as preclinical trials suggest that tissue levels of drug is dependent on the type of excipient used.^14^, ^15^ Furthermore, a previous observational study has shown that PCB with different excipients are associated with different risk of restenosis.^5^ Together, these results emphasize the importance of comparing assorted devices. In present study we show that a paclitaxel DCB using urea as an excipient, possess similar efficacy compared with other contemporary DCB (Table 3; Figure 2). The results were consistent when comparing the PCB with urea with other PCB (Figure S4). Paclitaxel is an effective drug alternative for DCB because of its high lipophility, resulting in an effective drug transfer to the vessel wall. Despite the recent emergence of sirolimus DCB (SCB), PCB remain the most common DCB type worldwide and in Sweden. In this trial, PCB was used in 97.0% of all procedures (Table S3). A recent meta‐analysis found greater minimum lumen diameter (weighted mean difference 0.10, 95% CI 0.02–0.17) at 9 to 12 months follow‐up favoring PCB.^16^ For long‐term clinically driven outcomes, data comparing PCB and SCB remains scarce. As the use of DCB continues to increase with the emergence of new DCB devices, further studies are warranted to assess the efficacy of different types of DCB to achieve a reliable outcome after DCB PCI. The Prevail Global Study will provide further insights on the efficacy of the PCB with urea for in‐stent restenosis and de novo small vessel disease.^8^

The results of this study are in line with a previous small prospective single‐arm study investigating the use of the PCB with urea (n=50).^7^ In this trial the 1‐year event rate of target lesion revascularization with the PCB with urea was 6.0% compared with 4.6% in present study (Table 3). The present study further explores a wide range of clinically important outcomes at 2‐years. The 2‐year outcome after DCB use varies across previous studies as they assess different study populations. When used for in‐stent restenosis, the 2‐year event rate of target lesion revascularization after DCB was 15% in the RIBS IV trial and 14.5% in the DAEDALUS trial.^17^, ^18^ However, in trials where DCB was used for de‐novo lesions the 2‐year rate of target lesion revascularization was significantly lower, 3.1% in the REC‐CAGEFREE 1 trial and 5.2% in the RESTORE SVD trial.^19^, ^20^ In this study including a diverse study population with 28.0% in‐stent restenosis, 55.3% American heart association classification of B2/C lesions and 22.8% bifurcations, the 2‐year event rate of target lesion revascularization was 7.4% for the PCB with urea (Table 3). The event rates reported in our study are within the range of previously reported for exclusively in‐stent restenosis and those reported for de novo only populations. Given our mixed population, the outcomes appear clinically reasonable for this real‐world population. Reflecting contemporary clinical practice in Sweden, the use of intracoronary imaging was low for both de novo lesions (14.5%) and in‐stent restenosis (27.1%). Previous studies have demonstrated that, when used during PCI with drug‐eluting stents, intracoronary imaging improves stent sizing, apposition, and clinical outcomes.^21^, ^22^ Although direct evidence in the context of PCI with DCB is limited, it is reasonable to assume that greater use of intracoronary imaging could similarly enhance procedural optimization and patient outcomes.

The use of DCB have gained interest with numerous applications including when DES implantation is not feasible, when short‐duration dual antiplatelet therapy is required, or in cases involving bifurcations and in‐stent restenosis with multiple stent layers. Because we utilized a national registry representing a true all‐comers population, our study encompasses a wide range of patients and lesions treated with DCB. In fact, over 60% of patients presented with ACS, and the inclusion of over 10% of STEMI patients is significant. Despite a variety in clinical presentations, it is important to note that the study population reflect a cohort where DCB were used, and the proportion of large de novo lesions was low [906 (14.0%) of the total procedures] as most large de novo lesions were most likely treated with drug‐eluting stents. Our subgroup analysis investigated the use of the PCB with urea for several clinically important subgroups (Figure 3). These analyses showed a consistent result for the PCB with urea compared with other DCB for both target lesion revascularization and myocardial infarction. Of interest, for patients undergoing PCI with DCB and stent in the same vessel, the PCB with urea was associated with lower rates of myocardial infarction compared with other contemporary DCB. These findings need to be validated in future studies.

## LIMITATIONS

This study poses several limitations. First and most important: treatment strategies were not random. This is the most important limitation as the retrospective design carries an inherent risk of selection bias. PCB have been used in Sweden since 2009 and we believe the implementation of different types of PCB are similar. This may be reflected in the baseline demographics, demonstrating similar patient characteristics between the 2 groups. Center effects and operator experience were partly accounted for through the nationwide, multicenter design, which reduces the influence of individual operators or centers on the estimated treatment effects. SCB emerged to the Swedish market recently, and it is reasonable to believe that SCB are used in selected cases. The SCB group comprised of only 253 (4.4%) devices. Thus, the results of SCB should be interpreted carefully. Results from ongoing randomized trials are expected to provide more insights into the clinical effect of SCB. Furthermore, despite extensive information available in the SCAAR registry, the registry lacks some important procedural characteristics variables including bailout stenting and predilation success. In addition, lesion‐level angiographic characteristics, such as lesion length, minimum lumen diameter and percentage diameter stenosis are not available. Sensitivity analyses are post‐hoc and should be interpreted cautiously, as differences in baseline characteristics across devices may not be fully accounted for.

## CONCLUSIONS

This is the largest study to date investigating the use of a PCB with urea, in a broad, real‐world patient population. In this analysis evaluating 2‐year results, the PCB with urea had comparable outcomes with other contemporary DCB. These data further support the safety and efficacy of the PCB with urea.

## Sources of Funding

This study was supported by an unrestricted grant from Medtronic.

## Disclosures

SJ declare consulting fees from Medtronic. EF declare institutional research grant from Medtronic. KG declare institutional research grant from Medtronic. MG declare consulting fees from Medtronic and Boston scientific. MG declare lecture Honoria from Medtronic, Boston Scientific and Philips IGT. MG declare participation in advisory board for Medtronic, Boston Scientific and IGT. Remaining authors declare no support for the submitted work from any organization, no financial relationship in the past 3 years with any organization that might have an interest in the submitted work, and no other relationships or activities that could appear to have influenced the submitted work.

## Supporting information

Tables S1–S5
Figures S1–S4
