# Supplementary material for: Real‐World Usage of a Paclitaxel‐Coated Balloon With Urea Compared With Other Contemporary Drug‐Coated Balloons: A 2‐Year Analysis From SCAAR in Over 6000 Patients
Source: J Am Heart Assoc. 2026 May 29;15(11):e045621. doi: 10.1161/JAHA.125.045621 (PMC13315143; doi:10.1161/JAHA.125.045621)
Supplement: Supplementary file 1 — Tables S1–S5 Figures S1–S4 [file JAH3-15-e045621-s001.pdf]

SUPPLEMENTAL MATERIAL

Table S1. The TARGET Checklist

Item no.		Checklist item			Location reported	
**Abstract**						
1	a	Identify that the study attempts to emulate a target trial using observational data. State the study objectives and briefly summarize the specified target trial.			Page 1 (Background)	
	b	Report the data sources used for emulation.			Page 1 (Methods)	
	c	Summarize key assumptions, statistical methods, findings and conclusions.			Page 1 (Results and conclusion)	
**Introduction**						
2	Background	Describe the scientific background of the study and the gap in knowledge.			Page 4	
3	Causal question	Summarize the causal question.			Page 4	
4	Rationale	Describe the rationale for emulating a target trial with the available data. Cite randomized trials informing the design of the target trial if applicable.			Page 5	
**Methods**						
5	Data sources	Cite the data sources contributing to the analyses and for each one describe the following: original purpose, type, the geographic locations, setting and time-period. If relevant, describe how the data were linked or pooled.			Page 6	
6	**Target trial specification**<br>Specify the components of the target trial protocol that would answer the causal question.		7	**Target trial emulation**<br>Describe how the components of the target trial protocol were emulated with the observational data, including how all variables were measured or ascertained.	Page 9	Page 4-5
	**Eligibility criteria**			**Eligibility criteria**		
	a	Describe the eligibility criteria.	a	Describe how the eligibility criteria were operationalized with the data.	Page 9	Page 6

**Treatment strategies**		**Treatment strategies**		Page 9	Page 6
b	Describe the treatment strategies that would be compared.	b	Describe how the treatment strategies were operationalized with the data.		
**Assignment procedures**		**Assignment procedures**		Page 9	Page 6
c	Report that eligible individuals would be randomly assigned to treatment strategies and may be aware of their treatment allocation.	c	Describe how assignment to treatment strategies was operationalized with the data.		
**Follow-up**		**Follow-up**		Page 9	Page 7
d	Clarify that follow-up would start at time of assignment to the treatment strategies. Specify when follow-up would end.	d	Clarify that follow-up starts at the time individuals were assigned to the treatment strategies. Describe how the end of follow-up was operationalized with the data.		
**Outcomes**		**Outcomes**		Page 7	Page 7
e	Describe the outcomes.	e	Describe how the outcomes were operationalized with the data.		
**Causal contrasts**		**Causal contrasts**		Page 9	Page 7
f	Describe the causal contrasts of interest, including effect measures.	f	Describe how the causal contrasts were operationalized with the data, including effect measures.		
**Identifying assumptions**		**Identifying assumptions**		Page 9	Page 9
g	Describe assumptions that would be made to identify each causal estimand. Describe the variables, if any, related to these assumptions.	g.i	For each causal estimand, describe assumptions made to identify it, including assumptions regarding baseline confounding due to lack of randomization.		
		g.ii	Describe how the variables related to these assumptions were operationalized with the data		Page 8-9
**Data analysis plan**		**Data analysis plan**		Page 8	Page 8

	h	For each causal estimand, describe the data analysis procedures and any associated statistical modelling assumptions, including approaches for handling missing data.	h.i	For each causal estimand, describe the data analysis procedures and any associated statistical modelling assumptions, including approaches for handling missing data.		
			h.ii	For each causal estimand, describe any additional analyses conducted to assess the sensitivity of the results to the choice of operationalizations, assumptions and analysis.		Page 8-9

**Results**			
**8**	Participant selection	Report numbers of individuals assessed for eligibility, eligible, and assigned to each treatment strategy. A flow diagram is strongly recommended.	Figure 1 (Page 28
**9**	Baseline data	Describe the distribution of characteristics of individuals at baseline, by treatment strategy.	Table 2 (Page 23-24)
**10**	Follow-up	Summarize length of follow-up and describe reasons for end of follow-up for each treatment strategy and causal contrast.	Page 7
**11**	Missing data	Describe the frequency of missing data in all variables, by treatment strategy when applicable.	Table 2 (Page 23-24)
**12**	Outcomes	Describe the frequency or distribution of each outcome, by treatment strategy.	Table 3 (Page 25)
**13**	Effect estimates	Report the effect estimates for each causal contrast with corresponding measures of precision, including both absolute and relative measures of effect, when applicable.	Table 3 (Page 25)
**14**	Additional analyses	Report results of all analyses to assess the sensitivity of the estimates to choices in operationalizations, assumptions and analysis.	Page 11-12
**Discussion**			
**15**	Interpretation	Provide an interpretation of the key findings.	Page 16
**16**	Limitations	Discuss the limitations of the study considering differences between the target trial and its emulation and the plausibility of assumptions, including assumptions regarding baseline confounding due to lack of randomization.	Page 16

Other information			
**17**	Ethics	Provide the institutional research board or ethics committee that approved the study and approval numbers, if relevant.	Page 6
**18**	Registration	State whether, when and where the study protocol was registered.	Page 6
**19**	Sharing of study materials	Provide information on whether data, analytic code and/or other materials are accessible, and where and how they can be accessed.	Page 6
**20**	Funding sources	Provide the sources of funding and detail the role of the funders in the design, conduct and reporting of the study.	Page 17
**21**	Conflicts of interest	State any conflicts of interest and financial disclosures for all authors.	Page 17

Citation: Cashin AG, Hansford HJ, Hernán MA, Swanson SA, Lee H, Jones MD, et al. Transparent Reporting of Observational Studies Emulating a Target Trial: The TARGET Statement. JAMA. 2025; DOI: 10.1001/jama.2025.13350
© 2025 Cashin et al. This is an Open Access article distributed under the terms of the Creative Commons Attribution-NoDerivatives License (CC BY-ND 4.0), which permits redistribution, commercial and non-commercial, provided the work is passed along unchanged and in whole, with credit to the original author(s).

Table S2. Outcome Definition

Outcome	Source	Level	Definition
All-cause mortality	National Population Registry	Procedure-level	All-cause death.
Myocardial infarction	RIKS-HIA	Procedure-level	Myocardial infarction was defined in accordance with the 4th universal definition of myocardial infarction. The outcome myocardial infarction is captured from RIKS-HIA and describes the hospitalization with MI diagnose (I21-I22) in any Swedish hospital. For individuals registered in RIKS-HIA there is an admission date and a discharge date. To avoid over-estimating myocardial infarction by defining a new registration as a new myocardial infarction when the two registrations in fact mirror that a patient might have been e.g. moved from one hospital to another, registrations with smaller gap than 2 days are counted as the same hospitalization, that is, as the same myocardial infarction. Periprocedural myocardial infarction was not included in the definition.

New revascularization with PCI	SCAAR	Procedure-level	Non-staged revascularization with any PCI, including revascularization of target vessel and non-target vessels.
Target lesion definite thrombosis	SCAAR	Device-level	Angiographically verified target lesion definite thrombosis.
Target lesion revascularization	SCAAR	Device-level	Non-staged repeat revascularization with PCI in the previously treated segment.
Target vessel revascularization	SCAAR	Device-level	Non-staged repeat revascularization with PCI in the previously treated vessel.
MACE	National Population Registry, RIKS-HIA and SCAAR	Procedure-level	Any target lesion revascularization, new myocardial infarction or all-cause mortality.

ICD = international classification of disease; PCI = Percutaneous coronary intervention; RIKS-HIA = Swedish Registry of Information and Knowledge about Swedish Heart Intensive Care Admissions; SCAAR = Swedish Coronary Angiography and Angioplasty Registry.

Table S3. Baseline Characteristics for SCB and Other PCB

**Procedure characteristics**	PCB with urea *N* = 1797	SCB *N* = 192	Other PCB *N* = 4470
Age	70.1 (61.8 – 77.9)	73.5 (66.3 – 78.8)	72.1 (63.1 – 78.5)
Female	399 (22.2%)	45 (23.4%)	971 (21.7%)
Smoking status: Non-smoker	756 (45.6%)	81 (45.8%)	1928 (45.2%)
Previous smoker	706 (42.6%)	79 (44.6%)	1833 (42.9%)
Current smoker	195 (11.8%)	17 (9.6%)	507 (11.9%)
Diabetes mellitus: Yes, insulin treated	240 (13.4%)	40 (20.9%)	651 (14.6%)
Yes, not insulin treated	328 (18.3%)	29 (15.2%)	787 (17.7%)
Yes, unknown treatment	3 (0.2%)	2 (1.0%)	12 (0.3%)
No	1218 (68.1%)	120 (62.8%)	3002 (67.4%)
Hyperlipidemia	1287 (71.8%)	150 (78.5%)	3281 (73.7%)
Hypertension	1416 (79.0%)	171 (89.1%)	3607 (81.0%)
Creatinine (μmol/L)	83.0 (70.2 – 100.0)	84.0 (69.0 – 102.8)	83.0 (71.0 – 99.0)
BMI (kg/m^2)	26.9 (24.4 – 29.8)	27.5 (25.0 – 30.8)	27.1 (24.4 – 30.1)
Previous MI	805 (45.0%)	94 (49.0%)	2138 (48.3%)
Previous PCI	1002 (55.8%)	130 (67.7%)	2660 (59.5%)
Previous CABG	191 (10.6%)	21 (10.9%)	454 (10.2%)
Indication: Stable CAD	434 (24.2%)	46 (24.0%)	1038 (23.2%)
Unstable angina	224 (12.5%)	28 (14.6%)	635 (14.2%)
NSTEMI	671 (37.3%)	21 (10.9%)	528 (11.8%)
STEMI	202 (11.2%)	79 (41.1%)	1756 (39.3%)
Other	266 (14.8%)	18 (9.4%)	513 (11.5%)
Angiographical finding: Non-conclusive	1 (0.1%)	0 (0.0%)	8 (0.2%)
Normal	98 (5.5%)	15 (7.8%)	345 (7.7%)
1-vessel not LM	752 (41.9%)	78 (40.6%)	1888 (42.2%)
2-vessel not LM	562 (31.3%)	58 (30.2%)	1219 (27.3%)
3-vessel not LM	281 (15.6%)	18 (9.4%)	702 (15.7%)
LM	102 (5.7%)	23 (12.0%)	308 (6.9%)
Successful procedure*	1774 (98.8%)	191 (99.5%)	4428 (99.1%)
**Device/lesion characteristics**	PCB with urea *N* = 2289	SCB *N* = 253	Other PCB *N* = 5438
Stent in same vessel	1108 (48.4%)	101 (39.9%)	2433 (44.7%)
IVUS used in same segment	411 (18.0%)	54 (21.3%)	658 (12.1%)
OCT used in same segment	121 (5.3%)	34 (13.4%)	348 (6.4%)
IVL used in same segment	34 (1.5%)	9 (3.6%)	98 (1.8%)
Atherectomy used in same segment	0 (0.0%)	0 (0.0%)	0 (0.0%)

Local success†	2174 (98.7%)	246 (98.0%)	5317 (98.4%)
Balloon length (mm)	20.0 (20.0 – 30.0)	20.0 (20.0 – 30.0)	20.0 (15.0 – 30.0)
Balloon diameter (mm)	2.5 (2.2 – 3.0)	3.0 (2.5 – 3.5)	2.5 (2.0 – 3.0)
Lesion type: De novo	1629 (71.2%)	134 (53.0%)	3507 (64.5%)
Other restenosis	19 (0.8%)	4 (1.6%)	76 (1.4%)
In-stent restenosis	641 (28.0%)	115 (45.5%)	1855 (34.1%)
B2/C	1265 (55.3%)	122 (48.2%)	2764 (50.8%)
Occlusion/CTO: Chronic total occlusion	75 (3.3%)	7 (2.8%)	193 (3.6%)
Acute total occlusion	162 (7.1%)	28 (11.1%)	531 (9.8%)
No occlusion	2050 (89.6%)	218 (86.2%)	4707 (86.7%)
Treated vessel: RCA	447 (19.5%)	46 (18.2%)	1059 (19.5%)
LM	36 (1.6%)	6 (2.4%)	120 (2.2%)
LAD	1166 (50.9%)	125 (49.4%)	2634 (48.4%)
LCx	600 (26.2%)	71 (28.1%)	1458 (26.8%)
Artery graft	6 (0.3%)	0 (0.0%)	3 (0.1%)
Vein graft	34 (1.5%)	5 (2.0%)	164 (3.0%)
Bifurcation	521 (22.8%)	45 (17.8%)	1081 (19.9%)

Presented as median (Q1-Q3) or n(%)

*Successful procedure. Subjective assessment by the operator. The operator has reached the main aim of the treatment.

†Treated stenosis of 50% or greater, with a reduction of at least 20% leading to a final stenosis degree of less than 50%, accompanied by good flow and no major complication.

BMI = body mass index; CABG = coronary artery by-pass graft; CAD = coronary artery disease; DCB = drug-coated balloons; IVL = intravascular lithotripsy; IVUS = intravascular ultrasound; LAD = left anterior descending; LCx = left circumflex artery; LM = left main; MI = myocardial infarction; NSTEMI = non-ST-segment elevation myocardial infarction; OCT = optical coherence tomography; PCB = paclitaxel-coated balloon; PCI = percutaneous coronary intervention; RCA = right coronary artery; SCB = sirolimus-coated balloon; STEMI = ST-segment elevation myocardial infarction.

Table S4. Number of Events and Kaplan-Meier Estimates for Subgroups

	1-year number of events (KM%)		2-year number of events (KM%)	
**Diabetes mellitus**	**PCB with urea**	**Other DCB**	**PCB with urea**	**Other DCB**
MACE	64 (13.8%)	278 (15.0%)	77 (24.1%)	348 (24.2%)
All-cause mortality	32 (6.5%)	130 (6.6%)	41 (14.1%)	163 (10.9%)
New myocardial infarction	20 (5.0%)	108 (6.9%)	23 (7.7%)	143 (11.6%)
New revascularization with PCI	47 (11.4%)	209 (12.2%)	58 (20.0%)	251 (18.0%)
Target lesion definite thrombosis	2 (0.4%)	11 (0.4%)	4 (1.3%)	15 (0.9%)
Target lesion revascularization	29 (5.5%)	137 (6.3%)	37 (10.7%)	162 (9.1%)
Target vessel revascularization	45 (8.3%)	196 (8.9%)	58 (17.2%)	234 (13.0%)
**Acute coronary syndrome**	**PCB with urea**	**Other DCB**	**PCB with urea**	**Other DCB**
MACE	115 (13.2%)	478 (12.6%)	136 (20.5%)	592 (20.0%)
All-cause mortality	54 (5.7%)	226 (5.9%)	61 (8.7%)	278 (9.4%)
New myocardial infarction	32 (4.1%)	179 (5.4%)	42 (7.3%)	236 (9.2%)
New revascularization with PCI	83 (10.4%)	362 (10.2%)	102 (17.0%)	444 (15.8%)
Target lesion definite thrombosis	6 (0.5%)	32 (0.7%)	9 (1.2%)	47 (1.6%)
Target lesion revascularization	60 (6.1%)	262 (6.2%)	73 (10.1%)	325 (9.9%)
Target vessel revascularization	82 (8.3%)	336 (7.7%)	100 (13.8%)	421 (12.8%)
**De novo lesions**	**PCB with urea**	**Other DCB**	**PCB with urea**	**Other DCB**
MACE	87 (7.9%)	369 (9.3%)	106 (13.1%)	461 (15.2%)
All-cause mortality	46 (4.0%)	192 (4.7%)	54 (6.4%)	235 (7.6%)
New myocardial infarction	25 (2.5%)	117 (3.3%)	31 (4.0%)	160 (6.1%)
New revascularization with PCI	66 (6.5%)	271 (7.1%)	82 (11.4%)	341 (11.8%)
Target lesion definite thrombosis	2 (0.1%)	19 (0.5%)	3 (0.3%)	24 (0.8%)

Target lesion revascularization	32 (2.5%)	128 (2.9%)	42 (5.2%)	161 (4.8%)
Target vessel revascularization	53 (4.2%)	187 (4.0%)	66 (7.8%)	239 (7.0%)
**In-stent restenosis**	**PCB with urea**	**Other DCB**	**PCB with urea**	**Other DCB**
MACE	71 (18.9%)	304 (16.4%)	79 (25.4%)	380 (25.7%)
All-cause mortality	26 (6.2%)	104 (5.4%)	28 (8.4%)	137 (9.5%)
New myocardial infarction	22 (6.0%)	137 (8.1%)	29 (11.2%)	174 (12.7%)
New revascularization with PCI	55 (16.2%)	271 (15.7%)	66 (24.1%)	325 (22.8%)
Target lesion definite thrombosis	5 (0.9%)	20 (0.8%)	7 (1.9%)	32 (2.2%)
Target lesion revascularization	42 (9.7%)	216 (10.1%)	47 (12.5%)	266 (15.6%)
Target vessel revascularization	52 (12.2%)	265 (12.3%)	60 (16.6%)	324 (18.8%)
**Small vessel**	**PCB with urea**	**Other DCB**	**PCB with urea**	**Other DCB**
MACE	81 (7.8%)	342 (9.2%)	103 (12.7%)	422 (14.7%)
All-cause mortality	41 (3.7%)	179 (4.8%)	48 (6.1%)	217 (7.5%)
New myocardial infarction	22 (2.3%)	115 (3.4%)	31 (4.9%)	154 (6.2%)
New revascularization with PCI	63 (6.6%)	255 (7.1%)	74 (10.3%)	317 (11.5%)
Target lesion definite thrombosis	3 (0.2%)	14 (0.4%)	3 (0.2%)	20 (0.8%)
Target lesion revascularization	25 (2.2%)	107 (2.7%)	29 (3.1%)	132 (4.3%)
Target vessel revascularization	47 (4.2%)	177 (4.3%)	54 (6.4%)	218 (6.9%)
**DCB and stent in same vessel**	**PCB with urea**	**Other DCB**	**PCB with urea**	**Other DCB**
MACE	58 (7.4%)	261 (9.7%)	72 (12.7%)	328 (15.8%)
All-cause mortality	33 (4.0%)	128 (4.5%)	38 (5.9%)	156 (7.2%)
New myocardial infarction	13 (1.9%)	95 (4.0%)	20 (4.7%)	129 (7.3%)
New revascularization with PCI	49 (6.7%)	204 (7.8%)	59 (10.9%)	257 (12.7%)
Target lesion definite thrombosis	1 (0.1%)	14 (0.6%)	1 (0.1%)	22 (1.3%)

Target lesion revascularization	18 (2.2%)	102 (3.8%)	22 (3.6%)	129 (6.0%)
Target vessel revascularization	32 (3.8%)	151 (5.3%)	38 (6.0%)	197 (9.1%)
**Bifurcation**	**PCB with urea**	**Other DCB**	**PCB with urea**	**Other DCB**
MACE	31 (8.2%)	112 (8.5%)	34 (11.7%)	145 (14.4%)
All-cause mortality	16 (3.7%)	57 (4.2%)	18 (5.6%)	68 (6.2%)
New myocardial infarction	8 (2.3%)	41 (3.7%)	8 (2.3%)	60 (7.3%)
New revascularization with PCI	29 (8.4%)	90 (6.5%)	32 (11.6%)	114 (10.9%)
Target lesion definite thrombosis	0 (0.0%)	4 (0.4%)	0 (0.0%)	7 (0.9%)
Target lesion revascularization	10 (2.8%)	45 (3.4%)	11 (4.3%)	54 (4.8%)
Target vessel revascularization	15 (3.9%)	60 (4.3%)	17 (6.9%)	78 (7.2%)
**Multi-vessel disease**	**PCB with urea**	**Other DCB**	**PCB with urea**	**Other DCB**
MACE	54 (11.0%)	191 (11.6%)	62 (15.9%)	232 (18.0%)
All-cause mortality	24 (4.9%)	93 (5.8%)	26 (6.3%)	115 (9.3%)
New myocardial infarction	16 (3.3%)	73 (5.1%)	21 (6.1%)	91 (8.0%)
New revascularization with PCI	35 (7.8%)	127 (8.0%)	44 (14.0%)	157 (13.2%)
Target lesion definite thrombosis	2 (0.2%)	7 (0.4%)	2 (0.2%)	8 (0.5%)
Target lesion revascularization	27 (4.4%)	95 (4.6%)	31 (6.5%)	110 (6.8%)
Target vessel revascularization	35 (5.9%)	124 (6.2%)	39 (8.0%)	148 (9.6%)
**Large vessel de novo**	**PCB with urea**	**Other DCB**	**PCB with urea**	**Other DCB**
MACE	30 (15.3%)	109 (13.4%)	36 (16.7%)	137 (22.3%)
All-cause mortality	14 (6.9%)	43 (4.7%)	15 (8.1%)	56 (8.8%)
New myocardial infarction	10 (5.4%)	44 (6.4%)	11 (7.3%)	55 (10.0%)
New revascularization with PCI	20 (11.6%)	87 (11.7%)	28 (26.7%)	106 (18.2%)
Target lesion definite thrombosis	0 (0.0%)	8 (1.3%)	1 (1.2%)	9 (1.7%)

Target lesion revascularization	15 (6.9%)	56 (6.7%)	21 (16.9%)	71 (11.5%)
Target vessel revascularization	17 (8.0%)	64 (7.4%)	24 (19.0%)	84 (13.9%)
**Chronic total occlusion**	**PCB with urea**	**Other DCB**	**PCB with urea**	**Other DCB**
MACE	4 (8.8%)	13 (7.4%)	5 (17.1%)	20 (17.9%)
All-cause mortality	1 (1.8%)	4 (3.0%)	1 (1.8%)	7 (7.5%)
New myocardial infarction	0 (0.0%)	3 (1.4%)	1 (8.3%)	6 (6.2%)
New revascularization with PCI	3 (7.1%)	10 (5.3%)	4 (15.5%)	15 (14.1%)
Target lesion definite thrombosis	0 (0.0%)	0 (0.0%)	0 (0.0%)	0 (0.0%)
Target lesion revascularization	3 (5.4%)	12 (5.7%)	3 (5.4%)	14 (8.6%)
Target vessel revascularization	3 (5.4%)	16 (7.4%)	4 (13.3%)	20 (12.2%)
**STEMI**	**PCB with urea**	**Other DCB**	**PCB with urea**	**Other DCB**
MACE	24 (13.6%)	107 (13.6%)	25 (15.0%)	130 (20.3%)
All-cause mortality	19 (10.7%)	72 (8.6%)	19 (10.7%)	83 (11.7%)
New myocardial infarction	2 (1.4%)	28 (4.6%)	3 (3.1%)	40 (8.7%)
New revascularization with PCI	9 (5.2%)	53 (7.6%)	11 (9.0%)	70 (13.3%)
Target lesion definite thrombosis	0 (0.0%)	4 (0.6%)	0 (0.0%)	10 (2.4%)
Target lesion revascularization	8 (3.8%)	24 (2.5%)	8 (3.8%)	41 (7.6%)
Target vessel revascularization	9 (4.2%)	32 (3.5%)	10 (5.8%)	54 (10.0%)

DCB = drug-coated balloon; KM = Kaplan-Meier; MACE = major adverse cardiac events; PCI = percutaneous coronary intervention; STEMI = ST-segment elevation myocardial infarction.

Table S5. PCB with Urea Compared to the Other Individual PCB

1-year MACE	PCB with urea (KM%)	Other PCB (KM%)	Unadjusted HR (95% CI)	Adjusted* HR (95% CI)
PCB A	10.6%	11.7%	1.08 (0.82-1.43) p-value: 0.556	0.93 (0.70-1.23) p-value: 0.624
PCB B	10.6%	12.3%	1.13 (0.91-1.39) p-value: 0.276	1.00 (0.81-1.24) p-value: 0.993
PCB C	10.6%	8.8%	0.81 (0.60-1.09) p-value: 0.165	0.80 (0.59-1.08) p-value: 0.142
**2-year MACE**	**PCB with urea (KM%)**	**Other PCB (KM%)**	**Unadjusted HR (95% CI)**	**Adjusted* HR (95% CI)**
PCB A	16.4%	19.0%	1.10 (0.86-1.40) p-value: 0.434	0.97 (0.76-1.23) p-value: 0.779
PCB B	16.4%	19.1%	1.14 (0.94-1.38) p-value: 0.190	1.02 (0.84-1.24) p-value: 0.865
PCB C	16.4%	14.6%	0.83 (0.64-1.08) p-value: 0.159	0.81 (0.62-1.05) p-value: 0.109

In this analysis, PCB with urea was the reference group.

CI = confidence interval; HR = hazard ratio; KM = Kaplan-Meier; MACE = major adverse cardiac events; PCB = paclitaxel-coated balloon.

*Adjusted for: age, sex, diabetes mellitus, hypertension, hyperlipidemia, previous myocardial infarction, previous PCI, previous coronary artery by-pass graft surgery, indication (chronic coronary syndrome, unstable angina, non-ST-segment elevated myocardial infarction, ST-segment elevation myocardial infarction and other indications), angiographic findings (non-conclusive, no significant stenosis, 1 vessel disease, 2 vessel disease, 3 vessel disease and left main disease), bifurcation, chronic total occlusion, in-stent restenosis, B2/C lesions (according to American heart association classification), use of intravascular ultrasound, use of optic coherence tomography, DCB diameter and use of stent during PCI.

Figure S1. Residual Plots.

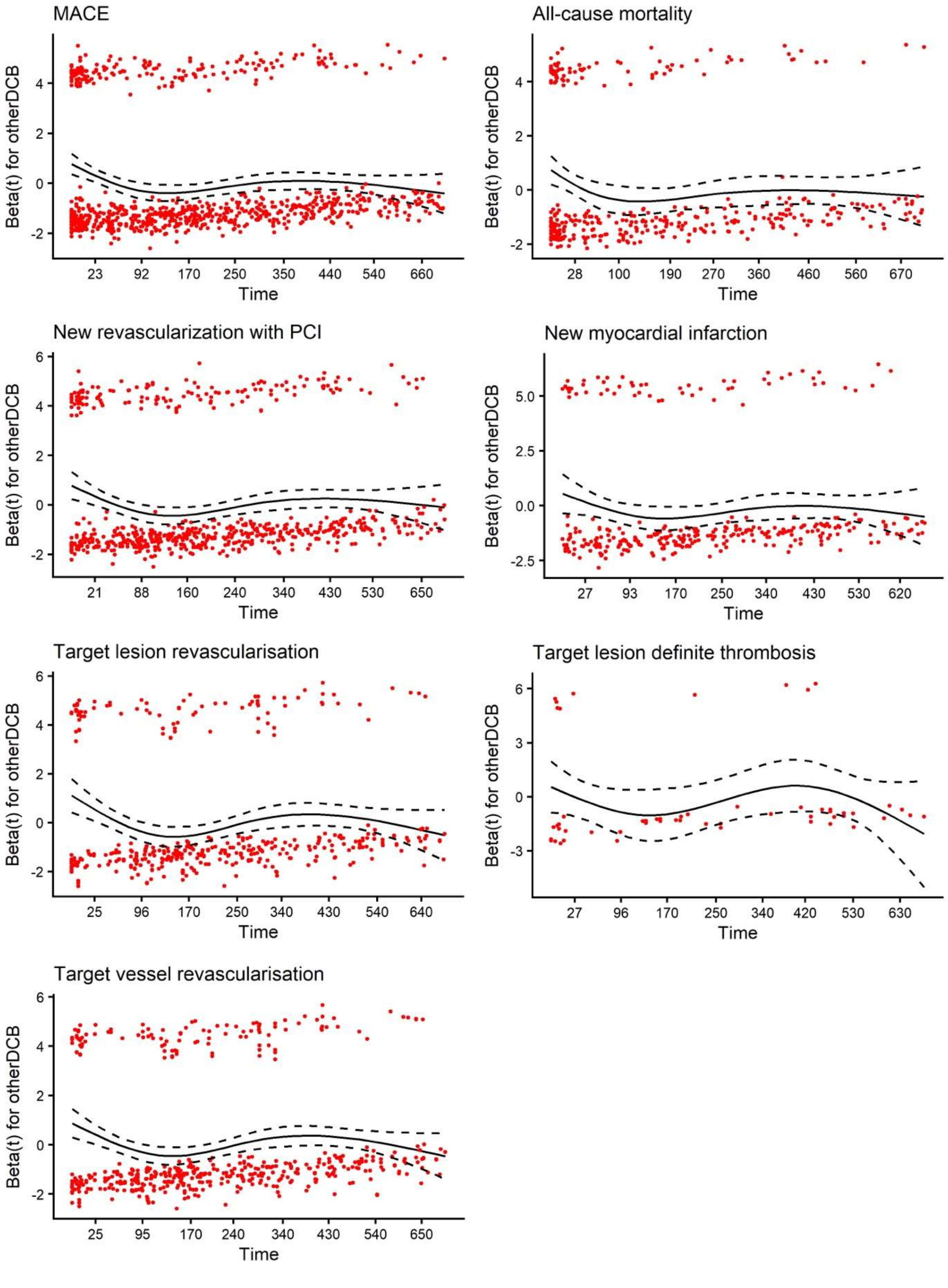

The proportional hazard assumption was tested using residual plots. The Schoenfeld residuals from adjusted models for checking of proportional hazards assumption are shown. The

residuals should be randomly scattered around zero and the smoothed line should be horizontal with no movement over time.

MACE = major adverse cardiovascular events; PCI = percutaneous coronary intervention.

Figure S2. Directed Acyclic Graph

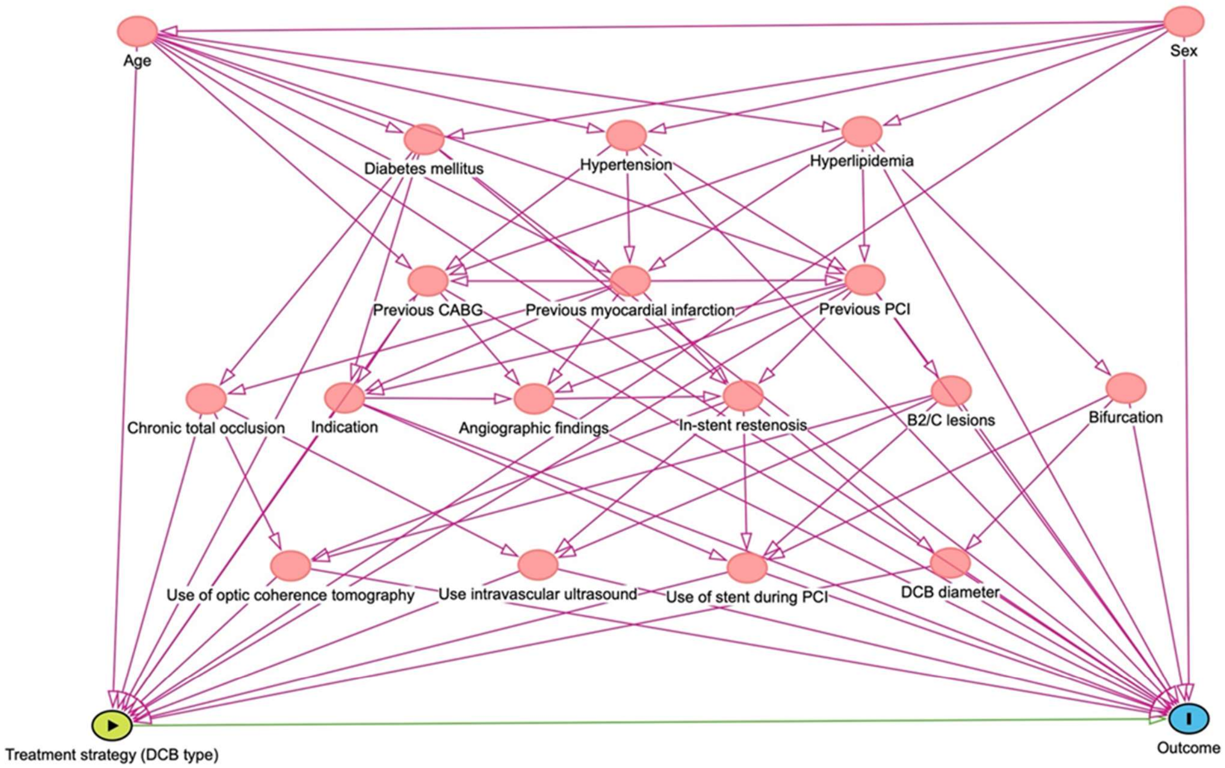

Graph illustrating a possible causal pathway of the variables included in the adjustment model. No colliders, mediators, or instrumental variables were included in the adjusted model.

DCB = drug-coated balloon; PCI = percutaneous coronary intervention.

Figure S3. Sensitivity Analysis.

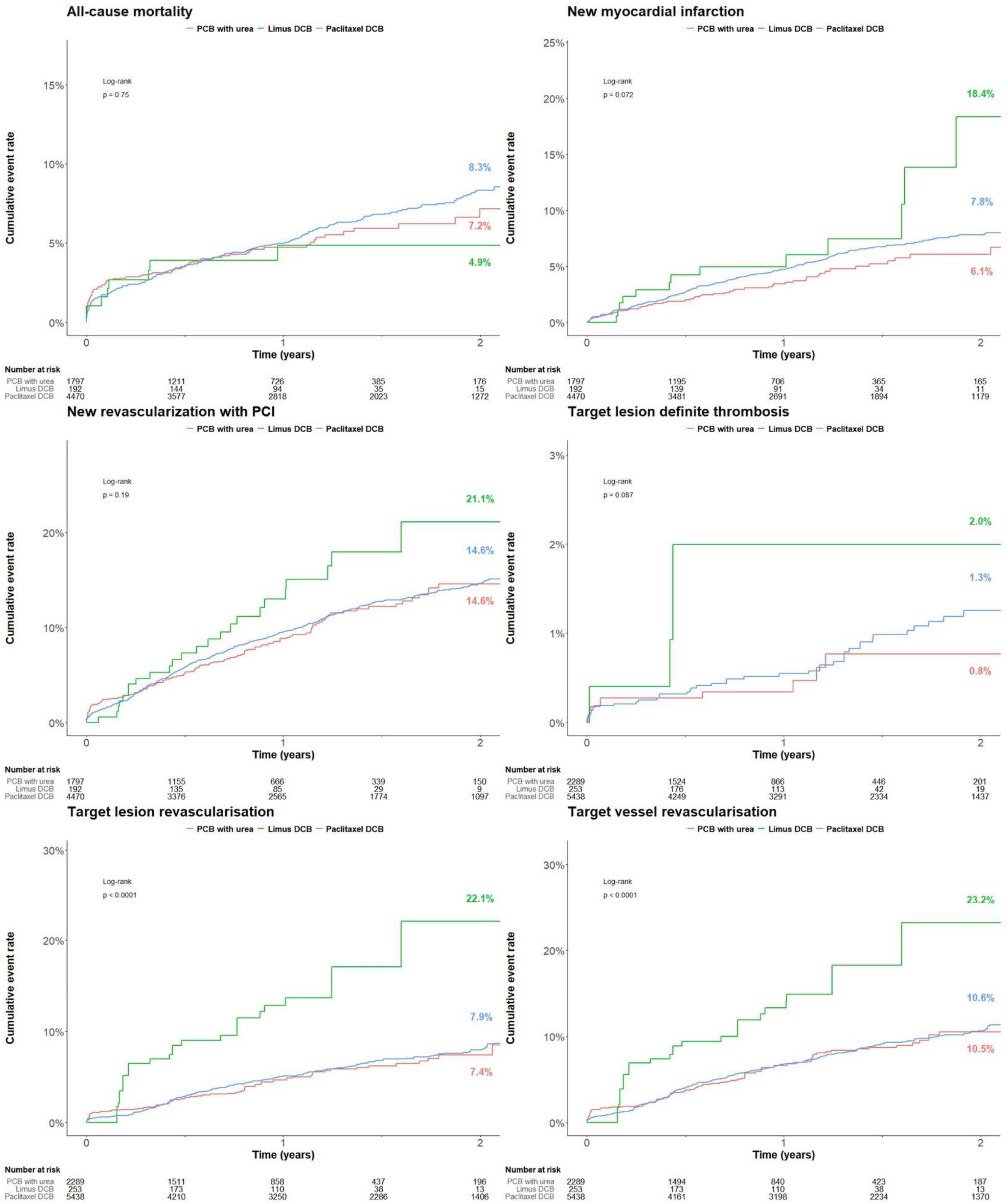

Illustrating the estimated event rates of all-cause mortality, new myocardial infarction and new revascularization with PCI for patients treated by the PCB with urea (red, N=1797

procedures), Other paclitaxel DCB (PCB) (blue, N=4470 procedures) and sirolimus DCB (SCB) (green, N=192 procedures); and target lesion definite thrombosis, target lesion revascularization and target vessel revascularization (N=2289 PCB with urea, N=5438 Other PCB and N=253 SCB).

PCB = paclitaxel-coated balloons; PCI = percutaneous coronary intervention; SCB = sirolimus-coated balloon.

Figure S4. PCB with Urea Compared to the Other Individual PCB

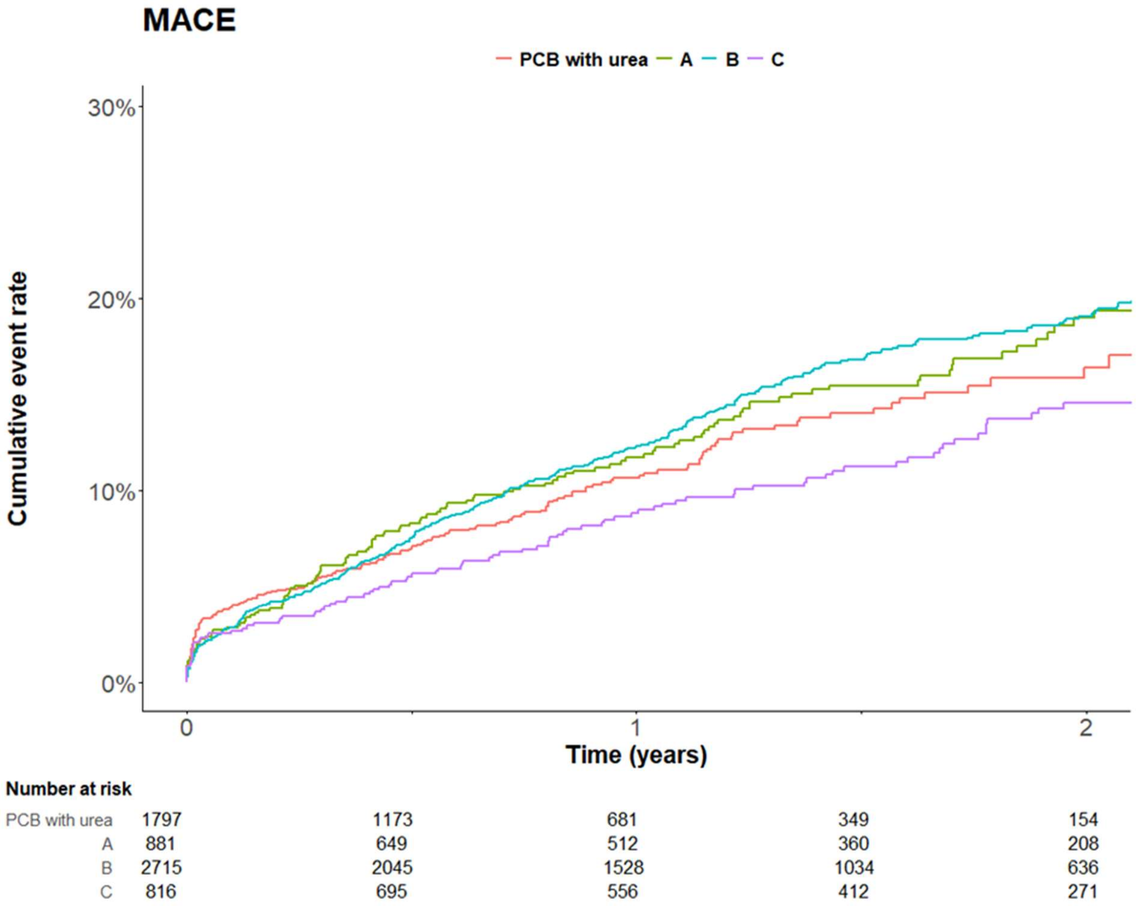

Kaplan-Meier plot illustrating the event rate of MACE for the PCB with urea and the individual other PCB.

MACE = major adverse cardiac events; PCB = paclitaxel-coated balloon.
